# SAMHD1 specifically restricts retroviruses through its RNase activity

**DOI:** 10.1186/s12977-015-0174-4

**Published:** 2015-06-02

**Authors:** Jongsu Choi, Jeongmin Ryoo, Changhoon Oh, Sungyeon Hwang, Kwangseog Ahn

**Affiliations:** Creative Research Initiative Center for Antigen Presentation, Seoul National University, Seoul, Republic of Korea; Department of the Interdisciplinary Program in Genetic Engineering, Seoul National University, Seoul, Republic of Korea; Department of Biological Sciences, Seoul National University, Seoul, Republic of Korea

**Keywords:** SAMHD1, RNase, Restriction factor, HIV-1, Retrovirus

## Abstract

**Background:**

Human SAMHD1 possesses dual enzymatic functions. It acts as both a dGTP-dependent triphosphohydrolase and as an exoribonuclease. The dNTPase function depletes the cellular dNTP pool, which is required for retroviral reverse transcription in differentiated myeloid cells and resting CD4^+^ T cells; thus this activity mainly plays a role in SAMHD1-mediated retroviral restriction. However, a recent study demonstrated that SAMHD1 directly targets HIV-1 genomic RNA via its RNase activity, and that this function (rather than dNTPase activity) is sufficient for HIV-1 restriction. While HIV-1 genomic RNA is a potent target for SAMHD1 during viral infection, the specificity of SAMHD1-mediated RNase activity during infection by other viruses is unclear.

**Results:**

The results of the present study showed that SAMHD1 specifically degrades retroviral genomic RNA in monocyte-derived macrophage-like cells and in primary monocyte-derived macrophages. Consistent with this, SAMHD1 selectively restricted retroviral replication, but did not affect the replication of other common non-retro RNA genome viruses, suggesting that the RNase-mediated antiviral function of SAMHD1 is limited to retroviruses. In addition, neither inhibiting reverse transcription by treatment with several reverse transcriptase inhibitors nor infection with reverse transcriptase-defective HIV-1 altered RNA levels after viral challenge, indicating that the retrovirus-specific RNase function is not dependent on processes associated with retroviral reverse transcription.

**Conclusions:**

The results presented herein suggest that the RNase activity of SAMHD1 is sufficient to control the replication of retroviruses, but not that of non-retro RNA viruses.

**Electronic supplementary material:**

The online version of this article (doi:10.1186/s12977-015-0174-4) contains supplementary material, which is available to authorized users.

## Background

Aicardi–Goutières syndrome (AGS) is an autoimmune encephalopathy caused by dysfunction of AGS-associated nucleases such as Trex1 and the RNase H2 complex [1, 2]. Naturally occurring mutations in AGS-related proteins are closely linked to inappropriate accumulation of intrinsic self-derived nucleic acids and aberrant sensing of nucleic acids, which triggers immune activation via up-regulation of type I interferon (type I IFN) signaling. This results in chronic inflammatory responses in the brain and skin [3]. In some cases, AGS is caused by mutations in the *samhd1* gene. In this context, it is hypothesized that the sterile alpha motif (SAM) and histidine-aspartic (HD) domain-containing protein 1 (SAMHD1) in humans might function as a nuclease that is involved in nucleic acid-mediated innate immunity [4].

SAMHD1 was first identified as a deoxyguanosine triphosphate (dGTP)-dependent deoxynucleotide triphosphohydrolase (dNTPase) [5], a function mediated entirely by the HD domain [6]. Moreover, the HD domain displays a wide variety of characteristics, all of which contribute to SAMHD1-protein interactions, SAMHD1 oligomerization [7], and nucleic acid binding [8, 9]. The dNTPase activity of SAMHD1 inhibits human immunodeficiency virus-type 1 (HIV-1) replication by cleaving and depleting cellular deoxyribonucleoside triphosphates (dNTPs) such that their levels are insufficient for retroviral reverse transcription (RT) [10–13]. However, the anti-retroviral mechanism mediated by SAMHD1 is limited to non-cycling cells such as macrophages, dendritic cells, and quiescent CD4^+^ T cells [14–17]. Although the phosphorylation status of SAMHD1 on residue T592 affects its anti-retroviral function [18], it does not interfere with its dNTPase activity [19, 20]. Taken together, these observations suggest that SAMHD1-mediated control of HIV-1 might not occur entirely in a dNTPase-dependent manner.

Recent studies show that SAMHD1 also acts as a nuclease and exhibits 3′–5′ exoribonuclease activity in vitro in a metal ion-dependent manner [21]. SAMHD1 preferentially cleaves single-stranded RNA, DNA substrates, and the RNA within DNA/RNA hybrids, suggesting that this function of SAMHD1 might be sufficient for participation in cellular nucleic acid metabolism and control of HIV-1 [21]. Consistent with this, we recently used AGS-causing SAMHD1 mutants to show that the RNase activity, but not the dNTPase activity, of SAMHD1 plays a crucial role in HIV-1 restriction by directly degrading intact HIV-1 genomic RNA [22]. The results suggested that specific targeting of HIV-1 RNA, rather than depletion of dNTPs, by SAMHD1 is necessary for HIV-1 clearance. Even though the in vivo and in vitro substrate specificity of SAMHD1 remains unclear, these previous studies suggest that SAMHD1 plays an important role in HIV-1 restriction and in the control of autoimmune responses.

The dNTPase activity of SAMHD1 has been intensively investigated in the context of retroviral restriction [6, 23]; however, it is not known whether the newly identified RNase activity of SAMHD1 has a unique ability to control HIV-1 infection or whether it can also control infection by other viruses. Given that SAMHD1 specifically targets HIV-1 RNA, it may also restrict other retroviruses that share common virological and biological features with HIV-1 (e.g., an RNA genome and RT). Here, we examined RNase-mediated retroviral restriction by SAMHD1. We found that, during infection by a panel of retroviruses, SAMHD1 specifically degraded retroviral genomic RNAs, thereby blocking productive infection. This indicates that the RNase activity of SAMHD1 is sufficient to control retroviral infection. Intriguingly, the antiviral ability of SAMHD1 was limited to retroviruses; it had no effect on non-retro RNA genome viruses. Furthermore, the retroviral-specific RNase activity of SAMHD1 was not dependent on progression of retroviral RT, implicating that SAMHD1 recognizes intact retroviral genomic RNAs at a very early time point following viral entry.

## Results

### SAMHD1 restricts a number of retroviruses by degrading genomic RNA

**Figure 1:**
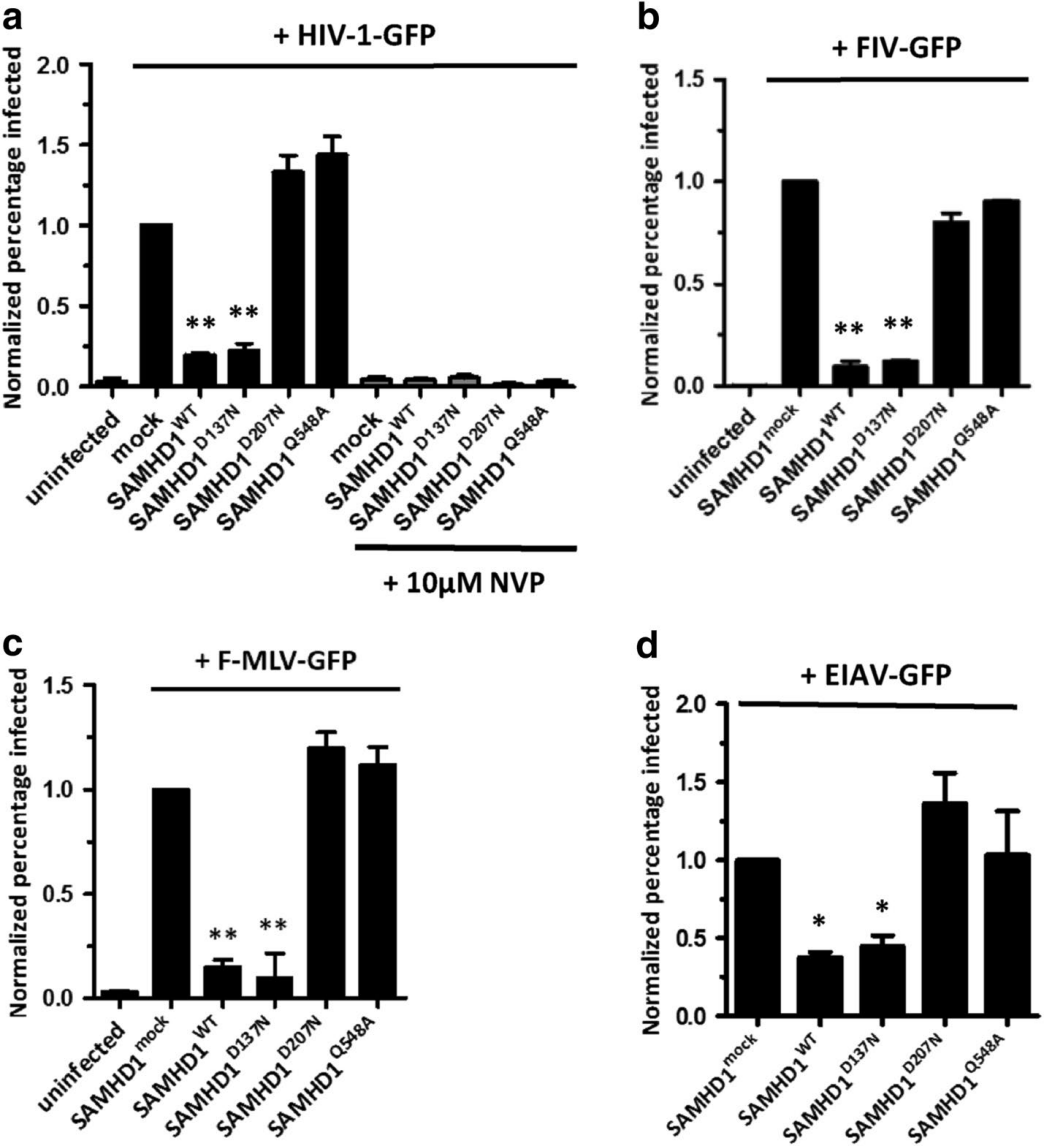
SAMHD1 restricts a variety of retroviruses through its RNase activity. **a** U937 cells stably expressing SAMHD1 mutants were differentiated with PMA. The cells were then pre-incubated with nevirapine for 24 h and then infected with HIV-1-GFP (60 ng of p24 per 6 × 10^5^ cells) for 2 h. The GFP-positive cells were examined by flow cytometry at 48 h post-infection. U937 cells pre-incubated with 10 μM nevirapine (NVP) for 24 h prior to HIV-1 transduction were used as a negative control. **b** PMA-treated U937 cells-expressing SAMHD1 mutants were infected with FIV-GFP at an MOI of 1. **c** SAMHD1 variants-expressing U937 cells were incubated in the presence of PMA and challenged with F-MLV-GFP at an MOI of 5. Reporter gene expression was analyzed by flow cytometry at 2 days post-infection. **d** PMA-treated U937 cells-expressing SAMHD1 mutants were differentiated with PMA and subsequently infected with EIAV-GFP at an MOI of 1. After 48 h, the infected cells were evaluated by flow cytometry. Values were normalized against the percentage of infected, mock-transfected cells. The data presented in **a**, **b**, **c**, and **d** are expressed as the mean ± SD of three independent experiments. *p < 0.05 and **p < 0.01 compared with the mock-transfected control (two-tailed Student’s *t* test).

The dual dNTPase and RNase functions of SAMHD1 play a role in its anti-retroviral function. Therefore, to examine the susceptibility of retroviruses to RNase-mediated control by SAMHD1, we used different retroviruses to infect U937 pro-monocytic cells stably expressing SAMHD1. In a previous study [22], we generated SAMHD1 mutants showing either dNTPase or RNase activity to identify the contribution of RNase activity to HIV-1 restriction. The corresponding SAMHD1-expressing U937 cells were then infected with VSV-G-pseudotyped reporter HIV-1, and the percentage of GFP-positive cells was evaluated by flow cytometry analysis (Additional file 1: Figure S1). Consistent with our previous study, we found that the RNase-positive SAMHD1^WT^ and the allosteric mutant SAMHD1^D137N^ (RNase-positive/dNTPase-negative) markedly reduced HIV-1 infectivity, whereas the catalytic mutant SAMHD1^D207N^ (dNTPase-negative/RNase-negative) inhibited HIV-1 restriction (Figure 1a; Additional file 1: Figure S1). SAMHD1^Q548A^ (dNTPase-positive) showed a similar infection ratio to SAMHD1^mock^ despite having the ability to deplete the intracellular dNTP pool (Figure 1a), suggesting that SAMDH1 acts as an HIV-1 restriction factor via its RNase activity rather than via its dNTPase activity as previously described [10–13]. Negative control cells (treated with 10 μM nevirapine (NVP), a non-nucleoside reverse transcriptase inhibitor) showed complete clearance of HIV-1 infection (Figure 1a; Additional file 1: Figure S1).

We next examined whether SAMHD1 inhibits other retroviruses via its RNase activity. Feline immunodeficiency virus (FIV), a non-primate lentivirus, has an RNA genome similar to that of other lentiviruses. In common with HIV-1, it also encodes several accessory proteins, including the Vif and Rev proteins. FIV possesses an additional small open reading frame, termed *orfA*, which codes for a factor that facilitates the transactivation of transcription during viral replication [24]. Unlike simian immunodeficiency virus (SIVsm) and HIV-2, FIV does not encode factors that counteract SAMHD1. Interestingly, SAMHD1 restricted FIV infection in an RNase activity-dependent manner. SAMHD1^D137N^ effectively prevented FIV transduction to almost the same extent as SAMHD1^WT^, whereas SAMHD1^D207N^ did not restrict FIV (Figure 1b). Moreover, SAMHD1^Q548A^ was sensitive to FIV infection (Figure 1b).

We also tested the RNase function of SAMHD1 by infecting cells with friend murine leukemia virus (F-MLV), which belongs to the gamma retrovirus family. MLV infects non-dividing cells inefficiently, and human TRIM5-α restricts MLV and EIAV infection during the viral uncoating process in the cytoplasm [23, 25]; however, this virus does not express viral factors that target SAMHD1 for degradation [23]. To overcome the low infectivity of MLV, we used F-MLV at a high MOI, resulting in a saturated viral infection (Additional file 2: Figure S2). In agreement with FIV restriction by SAMHD1, the RNase-mediated antiviral activity of SAMHD1 is necessary to control F-MLV infection. SAMHD1^WT^ or SAMHD1^D137N^ alone effectively reduced F-MLV infectivity (Figure 1c), while the catalytic mutant (D207N) and RNase-deficient (Q548A) SAMHD1 lost the ability to control the viral infection (Figure 1c).

Equine infectious anemia virus (EIAV), an ungulate lentivirus, encodes two auxiliary proteins known as Tat-TM fusion and S2 peptide [26–28]. These proteins are involved in disease pathogenesis but are not associated with SAMHD1 activity. As expected, only the RNase-active SAMHD1 variants were able to restrict EIAV infection in differentiated U937 cells (Figure 1d). These findings suggested that RNase activity of SAMHD1 is required for preventing retroviral infections via its RNase-mediated function.

**Figure 2:**
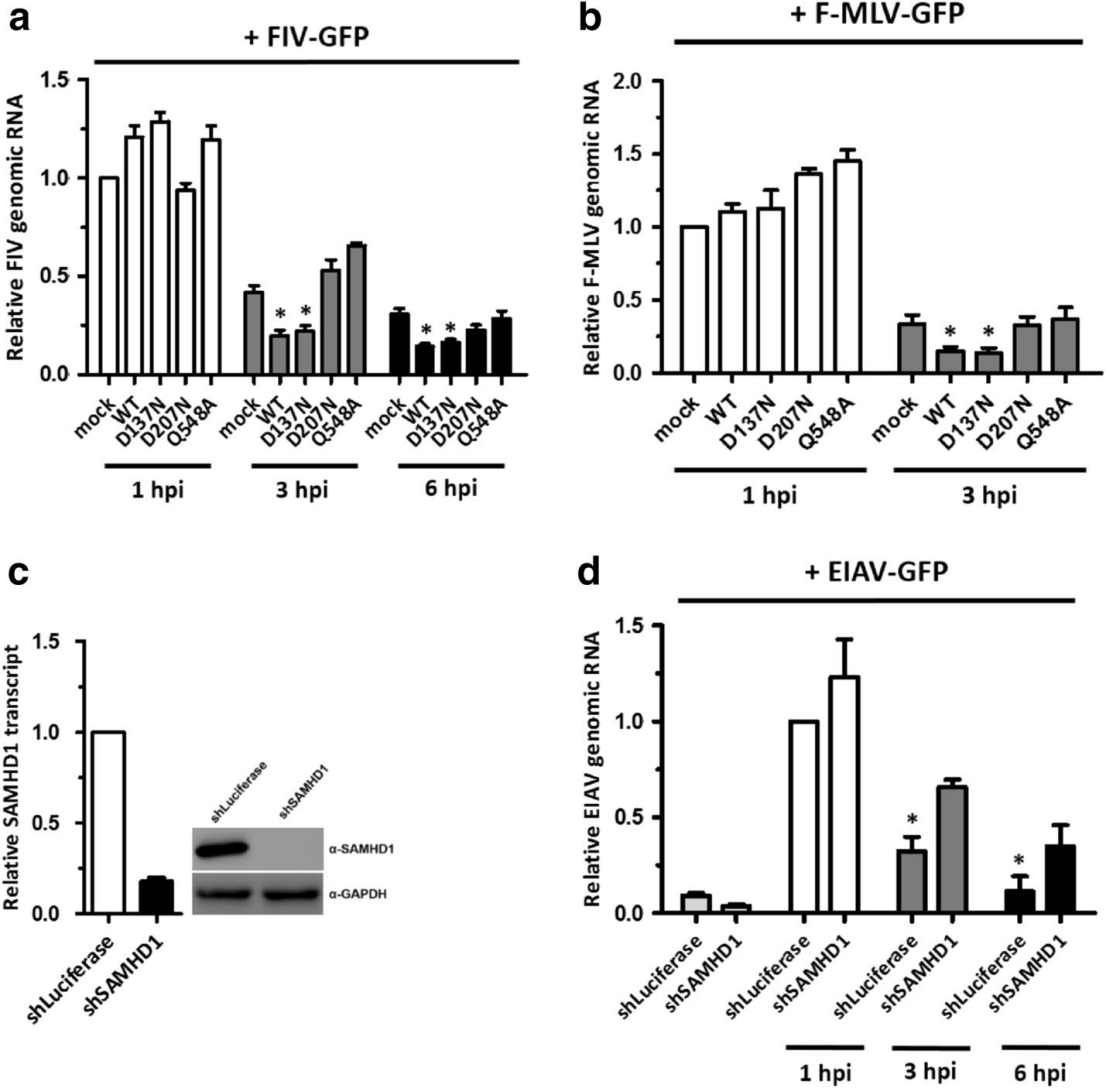
SAMHD1 affects the stability of retroviral genomic RNA. **a**, **b** PMA-treated U937 cells-expressing SAMHD1 mutants were infected with FIV-GFP at an MOI of 1 (**a**) or with F-MLV-GFP at an MOI of 5 (**b**) for 2 h. qRT-PCR was then performed with *gfp*-specific primers. **c**, **d** Immunoblot showing SAMHD1 expression (**c**). PMA-treated shLuciferase THP-1 and shSAMHD1 THP-1 cells were infected with EIAV-GFP at an MOI of 1 (**d**). qRT-PCR was then carried out with *gfp*-specific primers. Data were normalized against *β*-*actin* as an internal control. Data are expressed as the mean ± SD of three independent experiments in triplicate. *p < 0.05 compared with the mock-transfected control or the shLuciferase control at 1 h post-infection (two-tailed Student’s *t* test).

To further examine RNase-mediated SAMHD1 activity, we attempted to measure retroviral RNA levels at early time points following viral entry. We observed that SAMHD1-mediated FIV restriction was tightly associated with FIV genomic RNA levels following viral infection, supporting RNase-mediated control by SAMHD1. There was a significant reduction in SAMHD1-mediated FIV restriction only in the RNase-active SAMHD1-expressing cells at early time points (Figure 2a), indicating that the RNase activity of SAMHD1 mediates FIV restriction by degrading FIV genomic RNA. Consistent with this, SAMHD1-mediated control of F-MLV was directly linked to a reduction in F-MLV genomic RNA levels; a significant reduction in viral RNA levels was observed only in RNase-positive SAMHD1-expressing cells (Figure 2b), suggesting that SAMHD1 also controls F-MLV infection via its RNase-mediated function. Furthermore, depletion of SAMHD1 via expression of a stable shRNA (Figure 2c) led to a marked accumulation of EIAV genomic RNA at early time points post-infection (Figure 2d), suggesting that EIAV infection is also controlled by SAMHD1.

Since human primary monocyte-derived macrophages (primary MDMs) are an intact and biologically relevant system, we also examined the sensitivity of retroviruses to SAMHD1 in these cells. To do this, we treated freshly isolated primary MDMs with Vpx-containing virus-like particles (VLP) (Additional file 3: Figure S3A). As expected, silencing SAMHD1 via Vpx-mediated degradation [29] directly led to the accumulation of FIV genomic RNA at very early time points following viral transduction (Additional file 3: Figure S3B). Consistent with this, we also observed a reduction in the amount of F-MLV RNA (Additional file 3: Figure S3C) and EIAV genomic RNA (Additional file 3: Figure S3D) in active SAMHD1-expressing macrophages. Taken together, these results confirm that SAMHD1 controls a number of retroviral infections in an RNase activity-dependent, but not dNTPase-dependent, manner.

### The RNase-mediated antiviral activity of SAMHD1 is sufficient to inhibit production of diverse retroviral reverse transcripts

**Figure 3:**
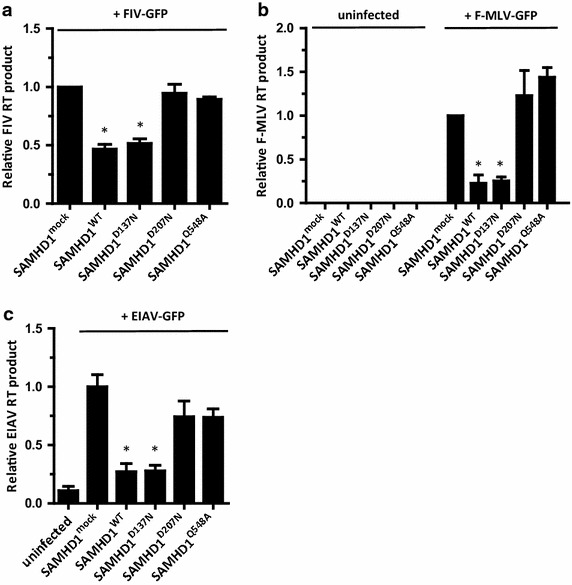
SAMHD1-mediated RNA degradation inhibits viral cDNA synthesis. Differentiated U937 cells-expressing SAMHD1 variants were infected with FIV-GFP (MOI = 1) (**a**), F-MLV-GFP (MOI = 5) (**b**), or EIAV-GFP (MOI = 1) (**c**) for 2 h. At 24 h post-infection, viral cDNA synthesis was monitored by qPCR using primers specific for *gfp* (**a**, **c**) or MLV late RT products (**b**). Data were normalized against an internal *mdm2* control. Data are expressed as the mean ± SD from three independent experiments in triplicate. *p < 0.05 compared with the mock-transfected control (two-tailed Student’s *t* test).

We next examined how SAMHD1-mediated RNA degradation affects the production of RT intermediates during retroviral infection. To do this, we measured the expression of retroviral RT products using quantitative PCR (qPCR). As shown in Figure 3a, the RNase activity of SAMHD1 led to an approximately 2-fold reduction in FIV cDNA synthesis at 24 h post-infection, demonstrating that RNase-mediated SAMHD1 function is required to prevent the production of viral cDNA intermediates. Consistently, only RNase activity-possessing SAMHD1 variants effectively blocked the production of F-MLV late reverse transcripts, whereas RNase-defective SAMHD1 mutants led to a robust increase in the amount of viral reverse transcripts at 24 h post-infection (Figure 3b). Moreover, SAMHD1 also inhibited EIAV transmission in the same manner. RNase-positive SAMHD1 variants impaired viral cDNA synthesis by 2-fold less than RNase-negative SAMHD1 mutants (Figure 3c). These results suggest that SAMHD1 functions during the early stages of retroviral infection, and that the degradation of retroviral genomic RNA abrogates the synthesis of viral cDNA intermediates.

We also determined whether SAMHD1-mediated RNA targeting has an effect on retroviral RT in primary MDMs. Viral RT intermediates were measured by qPCR at 24 h post-infection. Depleting SAMHD1 using Vpx-containing VLP increased the number of FIV cDNA copies (Additional file 4: Figure S4A). In agreement with this, SAMHD1 also inhibited the synthesis of F-MLV late RT products (Additional file 4: Figure S4B). Indeed, the production of RT intermediates by EIAV was also down-regulated only in the presence of RNase-positive SAMHD1 s (Additional file 4: Figure S4C). These findings suggested that SAMHD1 also prevents retroviral infection in MDMs through its RNase-mediated function. Taken together, these data demonstrate that SAMHD-mediated RNA degradation inhibits viral cDNA synthesis, thereby limiting retroviral replication.

### RNase activity of SAMHD1 is not required for the control of non-retro RNA viruses

**Figure 4:**
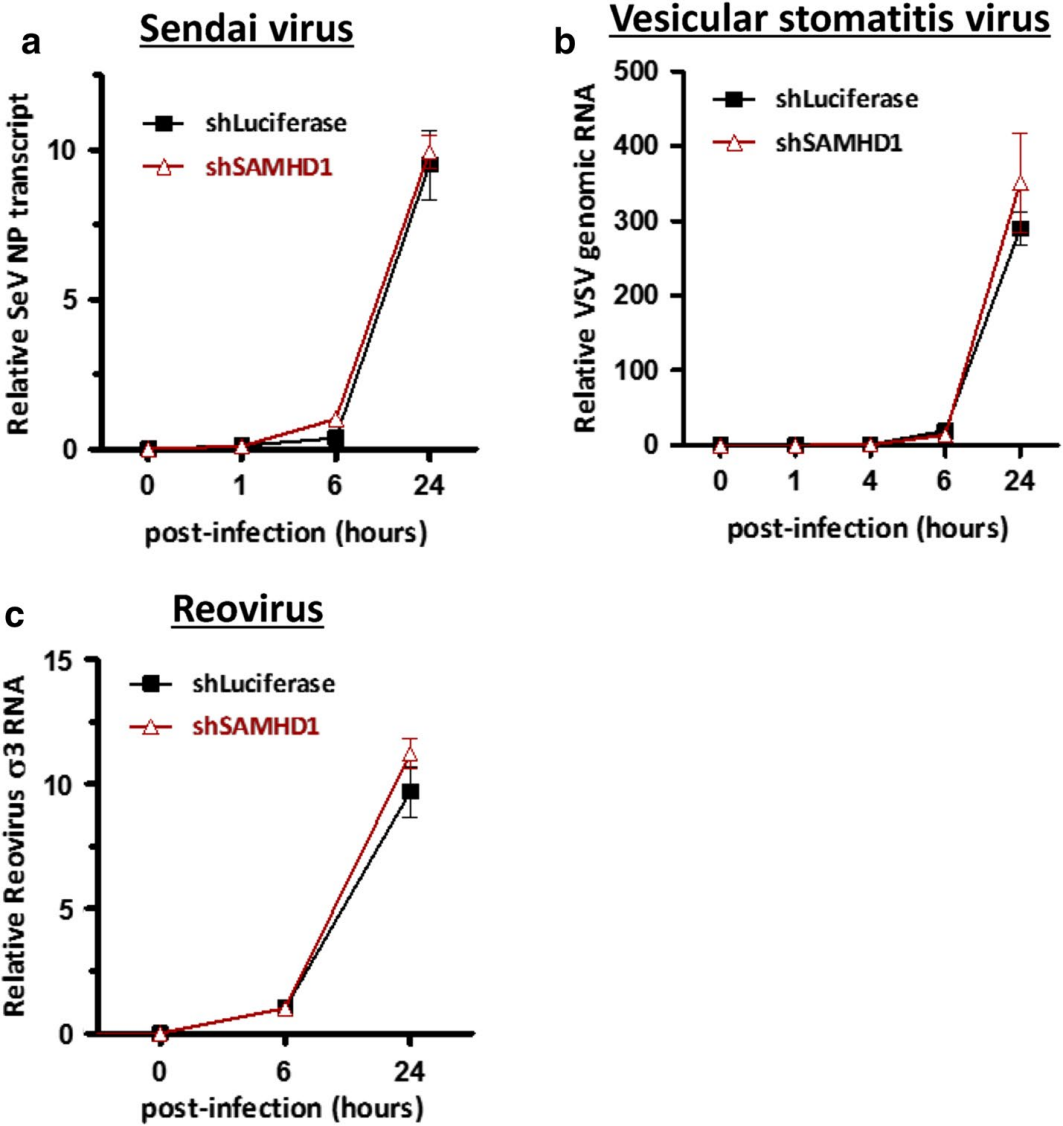
SAMHD1 does not inhibit the replication of non-retro RNA viruses. PMA-treated shLuciferase THP-1 and shSAMHD1 THP-1 cells were infected with SeV (**a**), VSV-GFP (**b**), or reovirus (**c**) at an MOI of 0.1. qRT-PCR was performed with primers specific for SeV nucleoprotein transcripts (**a**), *gfp* (**b**), or reoviral sigma 3 RNA (**c**). Data were normalized against *gapdh* as an internal control. Data are expressed as the mean ± SD of three independent experiments in triplicate.

We next examined whether the RNase activity of SAMHD1 also controls infection by non-retro RNA genome viruses. Given that most animal RNA viruses akin to retroviruses replicate outside the nucleus, we examined the sensitivity of a panel of common RNA viruses to SAMHD1. All viruses were chosen according to the polarity of their viral genomic RNA and their permissiveness to human-derived monocytic cells due to SAMHD1 activity in non-dividing cells. In principle, the RNA of these viruses is easily accessible in the cell cytoplasm during viral pathogenesis. Therefore, we sought to determine whether SAMHD1 affects the stability of the different viral RNA species. To achieve this, we infected SAMHD1-knockdown THP-1 cells (Figure 2c) with different RNA viruses. We then monitored RNA kinetics (as an indicator of viral replication) for up to 24 h. Sendai virus (SeV), also known as murine parainfluenza virus-type 1, is a non-segmented negative single-stranded RNA virus that has a very broad host spectrum. Since the negative SeV RNA genome replicates in the cytoplasm via an intermediary transcript with positive polarity (defined as the antigenome), we monitored antigenome RNA levels for 24 h (Figure 4a). There was a marked increase in SeV viral RNA levels, which peaked at 24 h post-infection; however, there was no significant accumulation of SeV RNA under SAMHD1-depleted conditions (Figure 4a). Thus, SAMHD1 does not interfere with SeV replication.

Vesicular stomatitis virus (VSV) is a negative single-stranded RNA virus with a broad host range. Like most negative ssRNA viruses, VSV shows a typical replication cycle. The viral nucleocapsid is released within the cytoplasm following virus attachment, penetration, fusion, and uncoating. As observed for SeV infection, VSV showed normal viral replication kinetics, which were independent of SAMHD1-expression (Figure 4b). This suggests that VSV also escaped restriction by SAMHD1.

Next, we tested the susceptibility of reovirus (*respiratory enteric orphan virus*) to SAMHD1. Reovirus is a segmented double-stranded RNA virus that also displays a broad host spectrum in animals. Because reovirus is able to replicate in the cytoplasm, we tried to measure σ3 transcript levels during viral infection; σ3 encodes a major structural component. We observed an increase in σ3 RNA levels at 24 h post-infection; however, the viral RNA kinetics in reovirus-infected THP-1 cells did not change after SAMHD1-depletion (Figure 4c). This result demonstrates that SAMHD1 does not affect the replication of reovirus. Taken together, these findings suggest that non-retro RNA viruses are not susceptible to the RNase activity of SAMHD1.

### Inhibiting HIV-1 RT does not affect the RNase activity of SAMHD1

**Figure 5:**
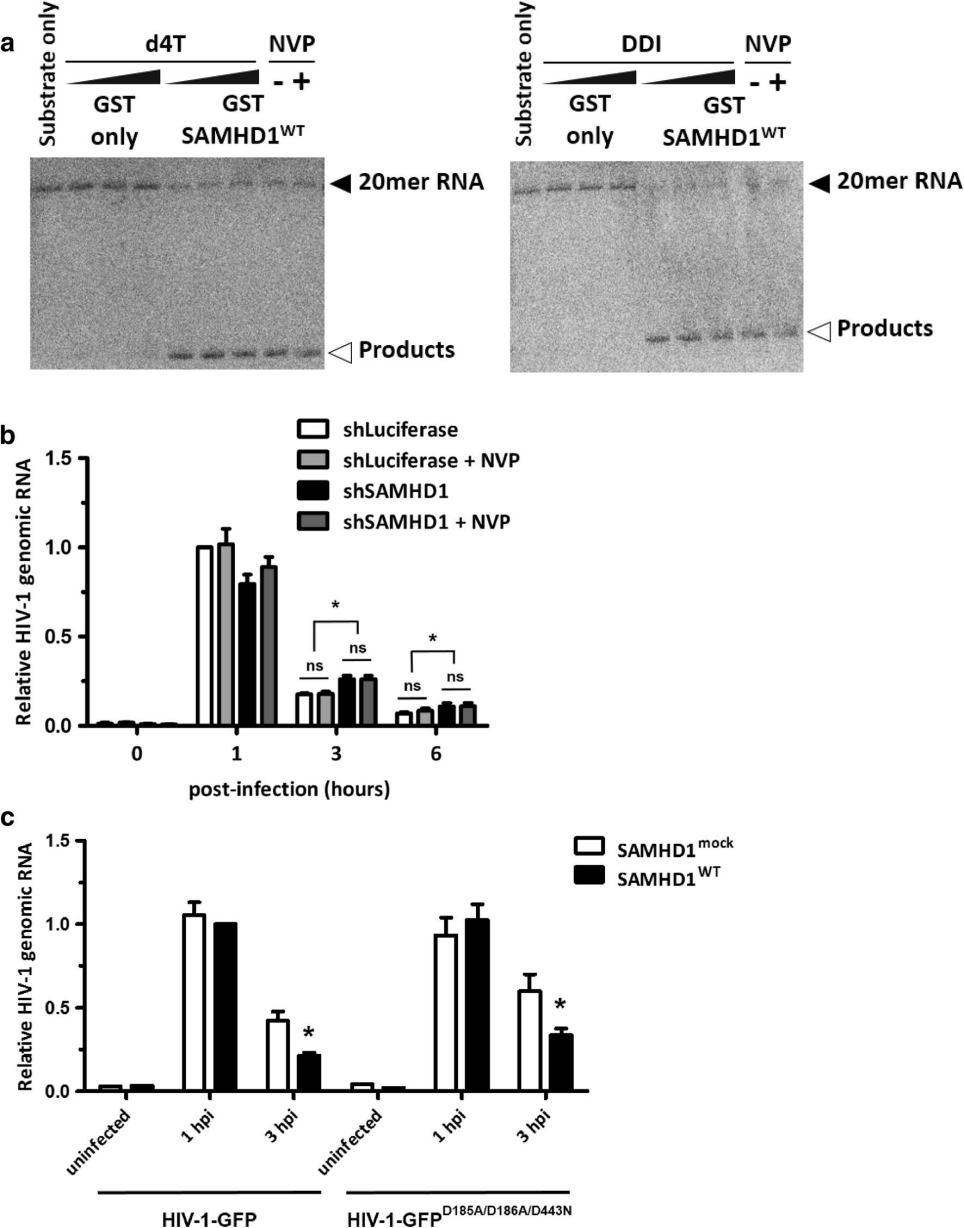
Inhibiting reverse transcription does not affect the RNase activity of SAMHD1. (**a**) An in vitro RNase assay was performed using 20mer ssRNA substrates. Purified GST-tagged SAMHD1^WT^ protein was pre-incubated with 0, 5, or 10 μM d4T (*left*) or DDI (*right*), or with 10 μM nevirapine. The reaction was performed in the presence of RNA substrates for 30 min at 37°C. The reaction was stopped by heating to 95°C for 10 min. **b** shLuciferase THP-1 and shSAMHD1 THP-1 cells were differentiated with PMA and then pre-incubated with nevirapine for 24 h, if required. The cells were then infected with HIV-1-GFP (60 ng per 6 × 10^5^ cells) for 2 h. At the indicated times, viral RNA levels were analyzed by qRT-PCR using *gfp*-specific primers. **c** Wild-type SAMHD1-expressing U937 cells were differentiated with PMA and then infected with HIV-1-GFP (10 ng per 1 × 10^5^ cells) or HIV-1^D185A/D186A/D443N^ (10 ng per 1 × 10^5^ cells). Viral RNA levels were monitored by qRT-PCR using *gfp*-specific primers. In **b**, **c**, data were normalized against an endogenous *β*-*actin* control. Data are expressed as the mean ± SD from three independent experiments in triplicate. *p < 0.05 compared with the shLuciferase control or the mock-transfected control at 1 h post-infection (two-tailed Student’s *t* test). *ns not significant.*

The RNase-mediated antiviral function of SAMHD1 appears to be limited to retroviruses. Because SAMHD1 directly targets retroviral genomic RNAs at early time points following infection, we examined whether retroviral RT, a hallmark of retroviral infection, is required for the retroviral specificity of SAMHD1. To do this, we first performed an in vitro nuclease assay to examine the RNase activity of SAMHD1 in the presence of reverse transcriptase inhibitors (RTIs). Although cellular dGTPs act as a substrate for, and an activator of, SAMHD1 dNTPase activity [10, 30], high levels of dGTP interfere with its RNase activity [20–22]. Unlike nucleoside analogue RTIs (NRTIs), which target the binding pocket within the reverse transcriptase active site, non-nucleoside RTIs (NNRTIs) allosterically bind to a site distant from the active site, known as the NNRTI pocket. Thus, we used these different types of RTI to exclude the possibility of SAMHD1 interference. We used two NRTIs (a thymidine analogue, d4T [2′,3′-didehydro-2′,3′-dideoxythymidine (also known as Stavudin)], and a guanosine analogue (DDI; 2′,3′-dideoxyinosine) and one NNRTI (nevirapine; NVP). Purified GST-tagged recombinant SAMHD1^WT^ was incubated with a synthetic nucleic acid substrate (20-mer) [22] in the presence of each RTI. As expected, SAMHD1^WT^ hydrolyzed single-stranded RNA; however, this activity was not affected by RTI treatment (Figure 5a). Consistent with this, HIV-1 RNA levels in differentiated THP-1 cells were not affected by SAMHD1-depletion, even though retroviral RT was completely blocked by addition of NVP (Figure 5b).

To exclude the possibility that viral RNA kinetics were affected by the RNase H activity of HIV-1 reverse transcriptase, we generated reverse transcriptase- and RNase H-defective HIV-1 (hereafter termed HIV-1^D185A/D186A/D443N^), in which D185 and D186 within the reverse transcriptase active site were mutated to alanine, and D443 was mutated to asparagine [31, 32]. Although blocking RNase activity during HIV-1 RT stabilizes HIV-1 viral RNA, we observed a marked decrease in HIV-1^D185A/D186A/D443N^ RNA levels in SAMHD1^WT^-expressing THP-1 cells (Figure 5c). Even though HIV-1^D185A/D186A/D443N^ infection of cells resulted in increased accumulation of viral RNA compared with that in cells infected with wild-type HIV-1 (Figure 5c), SAMHD1 still cleaved the viral RNA, indicating that SAMHD1 degrades HIV-1 RNA independently on HIV-1 RT. Taken together, these results indicate that retroviral RT does not determine the retroviral specificity of SAMHD1.

## Discussion

Given that SAMHD1, an intrinsic dNTP triphosphohydrolase, depletes cellular dNTP levels (which are required by reverse transcriptase for efficient synthesis of retroviral cDNA), it was believed that SAMHD1 would also inhibit infection by retroviruses other than HIV-1 [6, 23]. Even though the sensitivity of retroviruses to SAMHD1 differs slightly [23], suboptimal substrate conditions caused by SAMHD1 play a crucial role in limiting viral infections. It is, therefore, not surprising that SAMHD1 plays a role in inhibiting double-stranded DNA viruses such as herpes simplex virus 1 (HSV-1), vaccinia virus, and hepatitis B virus, all of which utilize cellular dNTPs as substrates for viral DNA polymerase [33, 34].

Contrary to previous studies that highlighted the dNTPase-mediated functions of SAMHD1, more recent studies show that SAMHD1 also displays 3′–5′ exoribonuclease activity [21]. Furthermore, this activity is necessary for HIV-1 regulation both in vitro and in vivo [22]. Here, we further examined the RNase activity of SAMHD1 by infecting cells with different retroviruses (FIV, F-MLV, and EIAV). Indeed, we also asked whether the RNase activity of SAMHD1 inhibits the replication of non-retro RNA genome viruses. The results support our previous data [22] showing that the RNase activity, rather than the dNTPase activity, of SAMHD1 inhibits a broad range of retroviruses by degrading their genomic RNA. Intriguingly, SAMHD1-mediated restriction occurred in a RT-independent manner, which is a hallmark of common retroviral pathogenesis. Because localization of SAMHD1 to the cytoplasm does not affect HIV-1 restriction [35, 36], our observations imply that SAMHD1 is able to recognize and target retroviral genomic RNA at early time points following virion uncoating.

Importantly, SAMHD1 did not influence the replication of non-retro RNA viruses such as SeV, VSV, and reovirus. Since replication of these common RNA viruses (like retroviruses) takes place entirely within the cytoplasm of the infected target cell, the viral genomic RNA/intermediates are easily exposed to attack by cellular ribonucleases. However, we found that unlike retroviral RNA, the stability of the non-retro viral RNA was not affected by SAMHD1 or SAMHD1-mediated dNTP depletion, suggesting that SAMHD1 selectively targets retroviral RNA species. A limitation of this experiment is that only three different RNA viruses were used to test SAMHD1-mediated antiviral activity; therefore, the discrepancy between “retro” viral and the “non-retro” viral restriction by SAMHD1 may need to be tested using other RNA viruses. However, the data clearly show that SAMHD1-mediated RNA digestion is not critical in some cases of non-retroviral infection.

It remains unclear how SAMHD1-mediated antiviral activity is restricted to retroviruses. Clearly, the single-round infection system used in the present study meant that well-known retroviral auxiliary proteins were not encoded; therefore, they cannot be involved in determining SAMHD1-mediated retroviral specificity. Since most cellular ribonucleases function in a sequence non-specific manner [37], and the group-specific antigen (*gag)* and *pol* genes (e.g., reverse transcriptase, RNase H, protease, and integrase) of most retroviruses share some similarities, it is possible that the role of SAMHD1 as a retroviral-specific RNase depends on other viral and/or cellular factors, which may enable SAMHD1 to recognize the crucial characteristics of retroviral RNA. Highly conserved secondary structure elements within the retroviral RNA genome may also be involved in recruiting SAMHD1.

In contrast to SAMHD1, other AGS-causing nucleases facilitate HIV-1 reverse transcription. The cytosolic nuclease, TREX1 (3′ repair exonuclease I), degrades excess single- and double-stranded nascent viral DNAs, thereby inhibiting their accumulation and preventing detection by the innate immune system [38]. The cellular RNase H2 complex in humans might also promote HIV-1 reverse transcription by cleaving RNA from RNA/DNA hybrids [37]. Although these enzymes play a different role in HIV-1 infection, they are closely linked in the context of cellular/viral nucleic acid pathways and in terms of AGS-related pathogenesis. Therefore, it might be interesting to explore the elements required for SAMHD1-mediated restriction in future studies. Studying the retroviral specificity of SAMHD1 will expand the spectrum of SAMHD1 activity beyond retroviral infection. Such insights may improve our understanding of SAMHD1-mediated nucleic acid pathways in the context of innate immunity.

## Conclusions

The RNase activity of SAMHD1 inhibits infection by several retroviruses, but not infection by a number of common non-retro RNA viruses. These results demonstrate that SAMHD1 selectively targets retroviral RNAs.

## Methods

### Plasmids

GFP-expressing reporter HIV-1 (hereafter referred to as HIV-1-GFP) does not encode any viral accessary proteins (*vif*^−^*vpr*^−^*vpu*^−^*env*^−^*nef*^−^). The GFP-expression cassette was inserted into *nef* as previously described [39]. The F-MLV-GFP [40] and the EIAV-GFP [41] and FIV-GFP vectors [25] have been described previously.

### Cells

Pro-monocytic human U937 and THP-1 cells were maintained in RPMI-1640 medium (Hyclone) supplemented with 10% fetal bovine serum (Hyclone). HeLa, Vero, and 293T cells were grown in Dulbecco’s modified Eagle’s medium (DMEM; Hyclone) supplemented with 2–10% fetal bovine serum, 10,000 Units/mL penicillin/streptomycin (Gibco), and GlutaMAX-I (Gibco). Cells were incubated at 37°C under a 5% CO_2_ atmosphere. Human primary monocyte-derived macrophages (primary MDMs) were isolated from fresh peripheral blood mononuclear cells (PBMCs) of healthy donors by immunomagnetic CD14-based selection (BD Biosciences), according to manufacturer’s instructions. Purified CD14^+^ monocytes were differentiated for 3 days in the presence of granulocyte-macrophage colony-stimulating factor (GM-CSF; 10 ng/mL) and macrophage colony-stimulating factor (M-CSF; 20 ng/mL), as described previously [22].

### Mutagenesis

HIV-1-GFP vector was used for generating HIV-1^D185A/D186A/D443N^ by site-directed mutagenesis as previously described [32]. The mutations were confirmed by DNA sequencing (Cosmogenetech co, Ltd., Seoul, South Korea).

### Retroviral stocks and virus infections

Retroviral and lentiviral stocks were prepared by standard polyethylenimine (PEI)-mediated transfection of 293T monolayers with Gag-Pol-encoding vectors (p5349, pFP93, and pEV53D for F-MLV, FIV, and EIAV, respectively), transfer vectors carrying virus-derived genomes bearing a GFP-expression cassette (p13077, pGiNW, and pEIAV-SIN6.1 CGFPW for F-MLV, FIV, and EIAV, respectively), and pMD.G at a ratio of 2:2:1. VSV-G-pseudotyped HIV-1-GFP or HIV-1^D185A/D186A/D443N^ virions were produced by cotransfecting 293T cells with HIV-1-GFP or HIV-1^D185A/D186A/D443N^ and pMD.G at a ratio of 5:1. Vpx-carrying VLPs and Vpx-depleted VLPs were generated by transfecting 293T monolayers with pSIV3 + and pSIV3 + Δ*vpx*, respectively. The cells were incubated for 4 h before the medium was replaced by fresh complete medium. Virion-containing supernatants were collected 48 h later and filtered through a 0.45 µm syringe filter. The concentration of HIV-1 p24 was measured in a p24 ELISA (QuickTiter Lentivirus Titer Kit; Cell Biolabs, Inc.) according to the manufacturer’s instructions. The multiplicity of infection (MOI) for F-MLV, FIV, and EIAV stocks was determined by transducing a known number of 293T cells with a known amount of virus particles and counting GFP-positive cells by flow cytometry. For virus challenge, U937 or THP-1 cells were seeded in 12- or 24-well plates at a density of 2–6 × 10^5^ cells mL^−1^ and differentiated into macrophage-like cells by overnight treatment with 30 ng mL^−1^ of phorbol 12-myristate 13-acetate (PMA). Viral infections were performed using a range of viral inocula (10 ng of HIV-1-GFP p24 per 10^5^ cells and F-MLV, FIV, and EIAV at an MOI of 1 or 5, as indicated). The inoculum was replaced with complete culture medium at 2 h post-infection. Infected cells were washed twice with PBS and harvested at the indicated time points. When required, cells were treated with a RTI (NVP, 10 μM) for at least 24 h before viral inoculation. For Vpx-mediated SAMHD1 silencing, cells were pre-incubated with Vpx-VLPs for 6 h before viral challenge, if required.

### RNA viruses and viral challenge

GFP-containing VSV (VSV-GFP) were propagated in HeLa cells, and reovirus (Type 3 Dearing strain) was propagated in Vero cells. Sendai virus was isolated from embryonated chicken eggs. Virus titers were determined in a standard plaque assay on HeLa cells or Vero cells. For infection with individual viruses, THP-1 cells (6 × 10^5^ cells mL^−1^) were seeded in 12-well plates and treated with PMA (30 ng/mL) overnight. The next day, 2% FBS-containing medium was added to the wells, and the cells were infected with virus at an MOI of 0.1. After allowing the virus to adsorb for 1 h, the medium was replaced with complete medium.

### Flow cytometry analysis

Differentiated U937 cells and THP-1 cells were detached using 1× PBS solution containing 10 mM EDTA and subsequently washed with 1× PBS solution. GFP expression in virus-infected cells was then evaluated by flow cytometry. Background fluorescence was measured in uninfected naïve cells and samples were acquired using a FACS Calibur flow cytometer (BD Biosciences). Data were analyzed using Cell Quest software (BD Biosciences).

### Isolation of genomic DNA and RNA

Genomic DNA was purified using a QIAamp® DNA Mini and Blood Mini Kit (Qiagen) according to manufacturer’s instructions. Total RNA was extracted using TRIzol® reagent (Ambion) according to the manufacturer’s instructions.

### Quantitative PCR (qPCR) and quantitative RT-PCR (qRT-PCR)

Equivalent amounts (50–100 ng) of purified genomic DNA from each sample were used for the qPCR reactions. For qRT-PCR, 1 μg of RNA was reverse-transcribed using the ReverTra Ace qPCR RT Kit (TOYOBO) according to the manufacturer’s recommendations. The cDNA was diluted with sterile deionized H_2_O (1 in 10), and qRT-PCR reactions were carried out in TOPreal qPCR PreMIX (Enzynomics) in the presence of 4 μL of diluted cDNA. The reaction volume was 20 μL, and all reactions were performed in triplicate. The PCR reactions were performed in the iCycler iQ real-time PCR detection system (BioRad). Data were normalized according to the expression of *β*-*actin*, *gapdh*, and *mdm2*, as indicated. Quantitative analyses of viral RNA transcripts and retroviral RT products were performed using the following primer-sets: SeV NP (forward, 5′-GGATCCCACGAATCGAGGTA-3′; reverse, 5′-TCCTGAAGATCTAAGGCAT-3′); reovirus σ3 (forward, 5′-GGGCTGCACATTACCACTGA-3′; reverse, 5′-CTCCTCGCAATACAACTCGT-3′); *gfp* (forward, 5′-CAACAGCCACAACGTCTATATCATG-3′; reverse, 5′-ATGTTGTGGCGGATCTTGAAG-3′); F-MLV late RT (forward, 5′-CGTCAGCGGGGGTCTTTC; reverse, 5′-CTGGGCAGGGGTCTCCCG) [23]; *gapdh* (forward, 5′-GCAAATTCCATGGCACCGT-3′; reverse, 5′-TCGCCCCACTTGATTTTGG-3′); *β*-*actin* (forward, 5′-AGAGCTACGAGCTGCCTGAC-3′; reverse, 5′-AGCACTGTGTTGGCGTACAG-3′); and *mdm2* (forward, 5′-GGTTGACTCAGCTTTTCCTCTTG -3′; reverse, 5′-GGAAAATGCATGGTTTAAATAGCC-3′).

### Protein expression and purification

For the in vitro nuclease assay, GST-tagged full-length human SAMHD1 (GST-SAMHD1^WT^) was expressed and purified from *E. coli* Rosetta (λDE3) (Novagen). Briefly, bacterial cells were grown at 37°C in Terrific broth containing ampicillin (100 μg mL^−1^) until the OD_600_ reached 2.0. The cells were then rapidly cooled to 16°C by incubation on ice. Following induction with 0.1 mM IPTG (Ducheba), the cells were incubated for 16 h at 16°C. The harvested bacterial pellet containing the GST fusion protein was lysed, and the protein was purified on a glutathione-Sepharose column as previously described [42].

### In vitro nuclease assay

Assays were performed in a 20 μL reaction volume as previously described [22]. Purified recombinant GST-tagged protein (150 nM) was added to reaction buffer supplemented with 5 mM MgCl_2_, 2 mM DDT, 10% glycerol, and 0.01% NP-40. If required, the reaction mixture was pre-incubated for 1 h at 37°C with a defined concentration of RTI. Following pre-incubation, ^32^P-labeled RNA substrates were added and incubated for 30 min at 37°C. The reaction was stopped by the addition of an equal volume of 2× formamide RNA loading buffer, followed by heating at 95°C for 10 min. The reaction products were separated in 15% polyacrylamide gels containing 7 M urea and buffered with 1× Tris–borate-EDTA. Gels were subjected to autoradiography and analyzed using a PhosphorImager (BAS2500; Fujifilm).

### Statistical analysis

Statistical analyses were performed using GraphPad Prism 5 (GraphPad Software). Comparisons between two groups were performed using two-tailed Student’s *t* test. Results were expressed as the mean ± SD, and *p* values <0.05 were considered statistically significant.

## Additional files

Additional file 1: Figure S1. The dNTPase activity of SAMHD1 is insufficient to control HIV-1 infection. U937 cells stably expressing wild-type or mutant SAMHD1 variants were differentiated by overnight incubation with PMA. The cells were then infected with HIV-1-GFP in the presence or absence of 10 μM nevirapine. The percentage of GFP-expressing cells was measured at 48 h post-infection. The result shown is representative of three independent experiments. Additional file 2: Figure S2. SAMHD1 restricts F-MLV infection through its RNase activity. Differentiated U937 cells-expressing mutant SAMHD1 protein were infected with F-MLV-GFP at an MOI of 5. At 48 h post-infection, the percentage of GFP-expressing cells was monitored by flow cytometry. The result shown is representative of three independent experiments. Additional file 3: Figure S3. SAMHD1 degrades retroviral genomic RNA in primary human MDMs. Human primary monocyte-derived macrophages (primary MDMs) were treated with Vpx-VLP (+Vpx-VLP) or with VLP control (–Vpx-VLP) for 6 h prior to infection with corresponding retroviruses. (A) SAMHD1 (upper panel) and GAPDH (lower panel) following VLP treatment was assessed by immunoblotting. (B, C, D) The cells were infected with the following VSV-G-pseudotyped retroviruses: FIV-GFP at an MOI of 1 (B), F-MLV-GFP at an MOI of 5 (C), or EIAV-GFP at an MOI of 1 (D). At corresponding time points, viral genomic RNA levels were determined by qRT-PCR using gfp-specific oligomers. Representative data were normalized to the amount of gapdh signal. Data are expressed as the mean ± S.D. from three independent experiments in triplicate. **p* < 0.05 compared with the Vpx-VLP-treated cells at 3 h post-infection (two-tailed 683 Student’s *t*-test). Additional file 4: Figure S4. SAMHD1 prevents retroviral cDNA synthesis in primary human MDMs. Freshly isolated primary MDMs were inoculated with Vpx-containing VLP (+Vpx-VLP) or with Vpx-depleted VLP control (–Vpx-VLP) for 6 h prior to viral challenge. Viral transduction was performed with the following: VSV-G-pseudotyped FIV-GFP (MOI = 1) (A), F-MLV-GFP (MOI 689 = 5) (B), or EIAV-GFP (MOI = 1) (C). At 24 h following viral infection, viral RT intermediates were quantified by quantitative PCR using primers specific for gfp (A, C) or MLV late RT (B). Data were normalized to the endogenous mdm2 signal. Graphs show the mean ± S.D. of three independent experiments (performed in triplicate). **p* < 0.05, ***p* < 0.01 (relative to +Vpx-VLP-transduced samples).

## Abbreviations

AGS: Aicardi–Goutières syndrome
EIAV: equine infectious anemia virus
FIV: feline immunodeficiency virus
F-MLV: friend murine leukemia virus
GFP: green fluorescent protein
GM-CSF: granulocyte–macrophage colony-stimulating factor
HIV-1: human immunodeficiency virus-type 1
IPTG: isopropyl-β-D-thiogalactopyranoside
M-CSF: macrophage colony-stimulating factor
PBMC: peripheral blood mononuclear cell
PMA: phorbol 12-myristate 13-acetate
MDM: monocyte-derived macrophage
RT: reverse transcription
SeV: Sendai virus
VLP: virus-like particles
VSV: vesicular stomatitis virus
VSV-G: vesicular stomatitis virus glycoprotein
