# Supplementary material for: SAMHD1 specifically restricts retroviruses through its RNase activity
Source: Retrovirology. 2015 Jun 2;12:46. doi: 10.1186/s12977-015-0174-4 (PMC4450836; doi:10.1186/s12977-015-0174-4)
Supplement: Additional file 1: Figure S1. — The dNTPase activity of SAMHD1 is insufficient to control HIV-1 infection. U937 cells stably expressing wild-type or mutant SAMHD1 variants were differentiated by overnight incubation with PMA. The cells were then infected with HIV-1-GFP in the presence or absence of 10 μM nevirapine. The percentage of GFP-expressing cells was measured at 48 h post-infection. The result shown is representative of three independent experiments. [file 12977_2015_174_MOESM1_ESM.pdf]

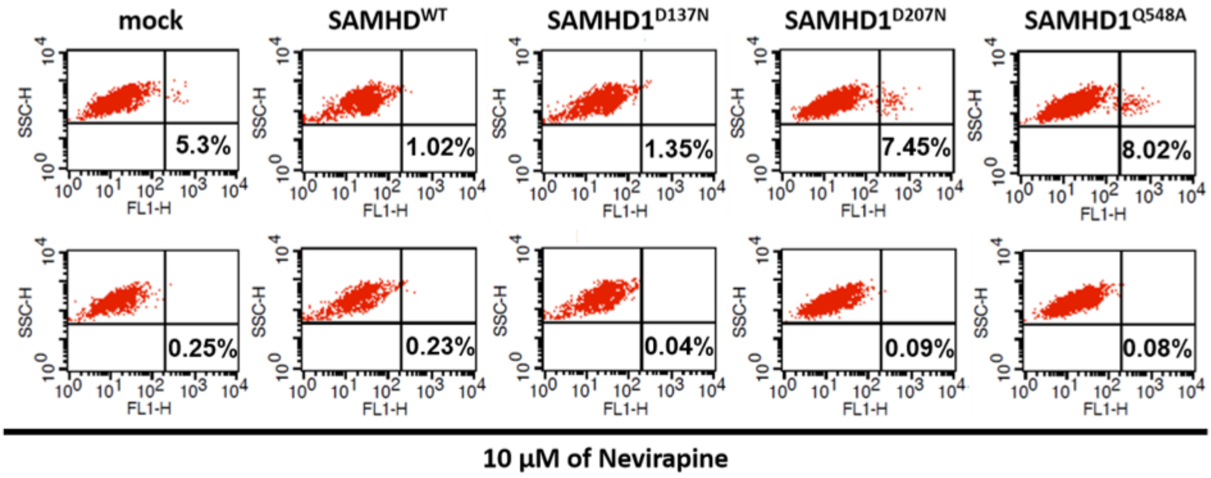

Additional file 1.

Figure S1.The dNTPase activity of SAMHD1 is insufficient to control HIV-1 infection. U937 cells stably expressing wild-type or mutant SAMHD1 variants were differentiated by overnight incubation with PMA. The cells were then infected with HIV-1-GFP in the presence or absence of 10 μM nevirapine. The percentage of GFP-expressing cells was measured at 48 h post-infection. The result shown is representative of three independent experiments.
