# Supplementary material for: SAMHD1 specifically restricts retroviruses through its RNase activity
Source: Retrovirology. 2015 Jun 2;12:46. doi: 10.1186/s12977-015-0174-4 (PMC4450836; doi:10.1186/s12977-015-0174-4)
Supplement: Additional file 2: Figure S2. — SAMHD1 restricts F-MLV infection through its RNase activity. Differentiated U937 cells-expressing mutant SAMHD1 protein were infected with F-MLV-GFP at an MOI of 5. At 48 h post-infection, the percentage of GFP-expressing cells was monitored by flow cytometry. The result shown is representative of three independent experiments. [file 12977_2015_174_MOESM2_ESM.pdf]

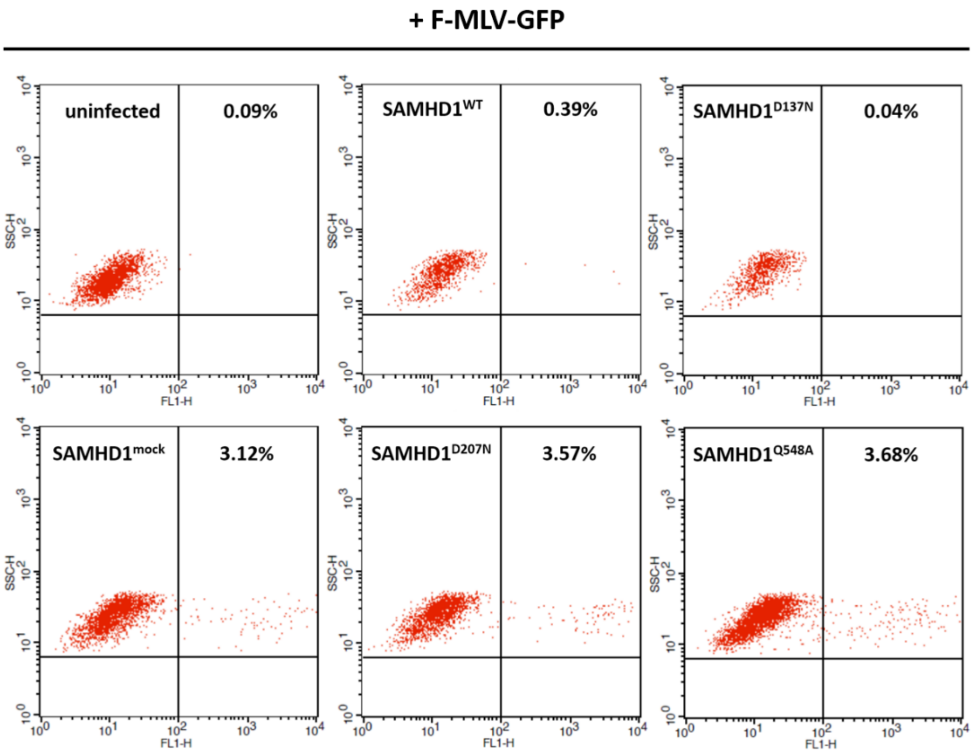

Additional file 2.

Figure S2.SAMHD1 restricts F-MLV infection through its RNase activity. Differentiated U937 cells-expressing mutant SAMHD1 protein were infected with F-MLV-GFP at an MOI of 5. At 48 h post-infection, the percentage of GFP-expressing cells was monitored by flow cytometry. The result shown is representative of three independent experiments.
