# Supplementary material for: SAMHD1 specifically restricts retroviruses through its RNase activity
Source: Retrovirology. 2015 Jun 2;12:46. doi: 10.1186/s12977-015-0174-4 (PMC4450836; doi:10.1186/s12977-015-0174-4)
Supplement: Additional file 3: Figure S3. — SAMHD1 degrades retroviral genomic RNA in primary human MDMs. Human primary monocyte-derived macrophages (primary MDMs) were treated with Vpx-VLP (+Vpx-VLP) or with VLP control (–Vpx-VLP) for 6 h prior to infection with corresponding retroviruses. (A) SAMHD1 (upper panel) and GAPDH (lower panel) following VLP treatment was assessed by immunoblotting. (B, C, D) The cells were infected with the following VSV-G-pseudotyped retroviruses: FIV-GFP at an MOI of 1 (B), F-MLV-GFP at an MOI of 5 (C), or EIAV-GFP at an MOI of 1 (D). At corresponding time points, viral genomic RNA levels were determined by qRT-PCR using gfp-specific oligomers. Representative data were normalized to the amount of gapdh signal. Data are expressed as the mean ± S.D. from three independent experiments in triplicate. *p < 0.05 compared with the Vpx-VLP-treated cells at 3 h post-infection (two-tailed 683 Student’s t-test). [file 12977_2015_174_MOESM3_ESM.pdf]

Figure S3. Choi *et al.*

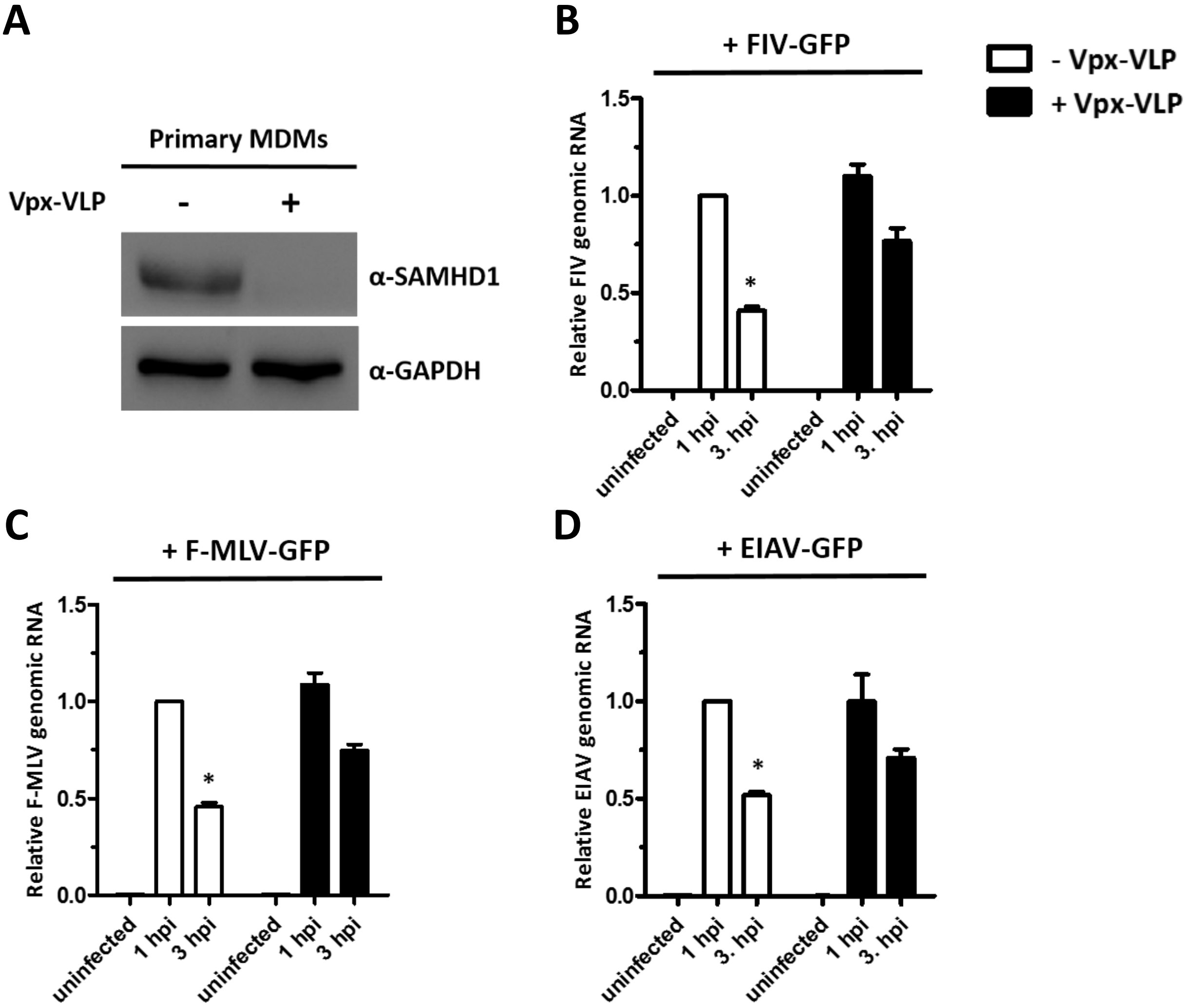

Additional file 3.

Figure S3.SAMHD1 degrades retroviral genomic RNA in primary human MDMs. Human primary monocyte-derived macrophages (primary MDMs) were treated with Vpx-VLP (+ Vpx-VLP) or with VLP control (−Vpx-VLP) for 6 h prior to infection with corresponding retroviruses. (A) SAMHD1 (upper panel) and GAPDH (lower panel) following VLP treatmentwas assessed by immunoblotting. (B, C, D) The cells were infected with the following VSV-G-pseudotyped retroviruses: FIV-GFP at an MOI of 1 (B), F-MLV-GFP at an MOI of 5 (C), or EIAV-GFP at an MOI of 1 (D). At corresponding time points, viral genomic RNA levels were determined by qRT-PCR using gfp-specific oligomers. Representative data were normalized to the amount of gapdh signal. Data are expressed as the mean ± S.D. from three independent experiments in triplicate. *$p < 0.05$ compared with the Vpx-VLP-treated cells at 3 h post-infection (two-tailed 683 Student’s t-test).
