# Supplementary material for: SAMHD1 specifically restricts retroviruses through its RNase activity
Source: Retrovirology. 2015 Jun 2;12:46. doi: 10.1186/s12977-015-0174-4 (PMC4450836; doi:10.1186/s12977-015-0174-4)
Supplement: Additional file 4: Figure S4. — SAMHD1 prevents retroviral cDNA synthesis in primary human MDMs. Freshly isolated primary MDMs were inoculated with Vpx-containing VLP (+Vpx-VLP) or with Vpx-depleted VLP control (–Vpx-VLP) for 6 h prior to viral challenge. Viral transduction was performed with the following: VSV-G-pseudotyped FIV-GFP (MOI = 1) (A), F-MLV-GFP (MOI 689 = 5) (B), or EIAV-GFP (MOI = 1) (C). At 24 h following viral infection, viral RT intermediates were quantified by quantitative PCR using primers specific for gfp (A, C) or MLV late RT (B). Data were normalized to the endogenous mdm2 signal. Graphs show the mean ± S.D. of three independent experiments (performed in triplicate). *p < 0.05, **p < 0.01 (relative to +Vpx-VLP-transduced samples). [file 12977_2015_174_MOESM4_ESM.pdf]

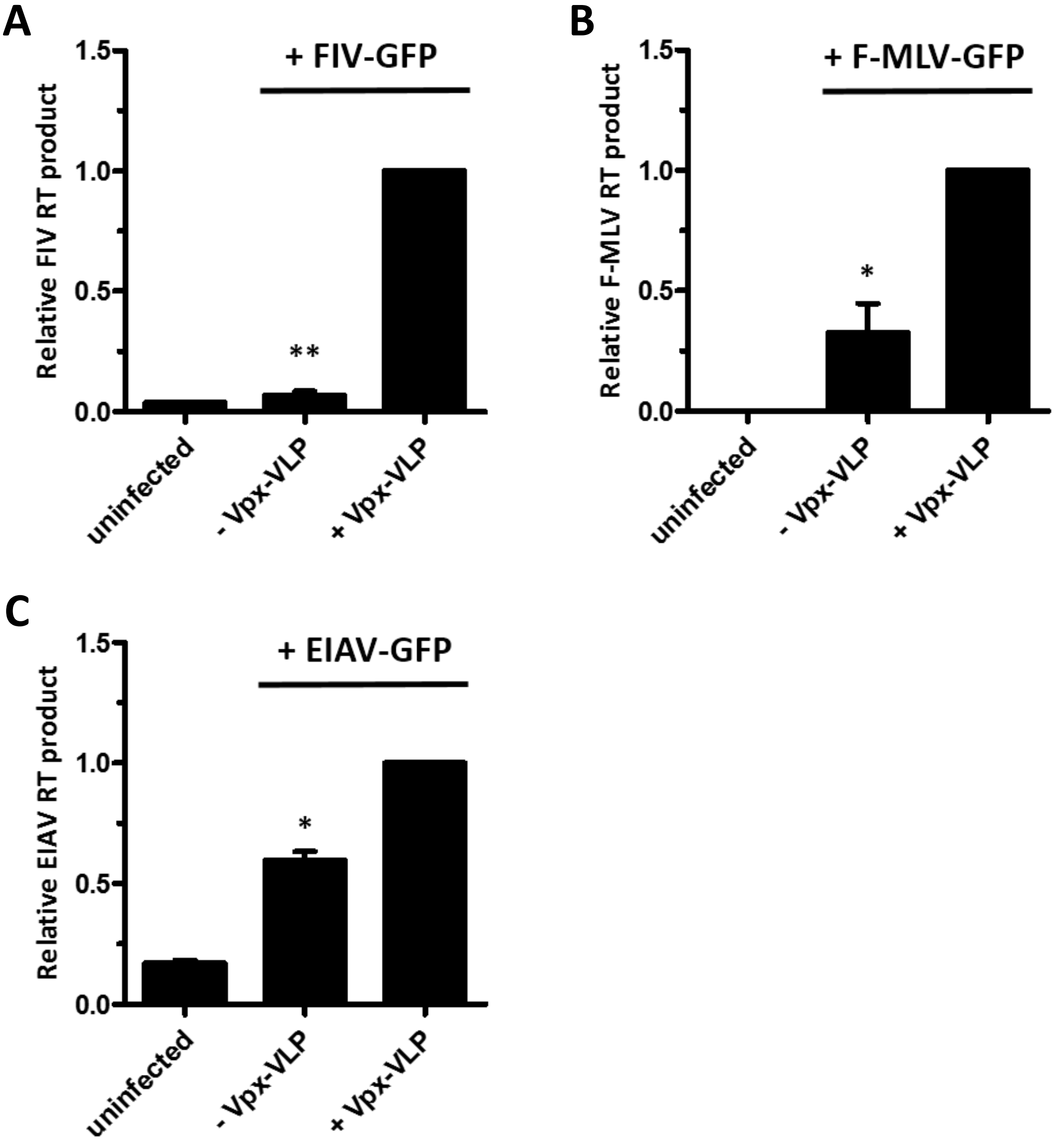

Additional file 4.

Figure S4.SAMHD1 prevents retroviral cDNA synthesis in primary human MDMs. Freshly isolated primary MDMs were inoculated with Vpx-containing VLP(+ Vpx-VLP)or with Vpx-depleted VLPcontrol (−Vpx-VLP) for 6 h prior to viral challenge. Viral transduction was performed with the following: VSV-G-pseudotyped FIV-GFP (MOI =1) (A), F-MLV-GFP (MOI 689 =5) (B), or EIAV-GFP (MOI =1) (C). At 24 h following viral infection, viral RT intermediates were quantified by quantitative PCR using primers specific for gfp (A, C) or MLV late RT (B). Data were normalized to the endogenous mdm2 signal.Graphs show the mean ± S.D. of three independent experiments (performed in triplicate). *p < 0.05, **p < 0.01 (relative to + Vpx-VLP-transduced samples).
